# Sociodemographic characteristics and their association with survival in women with cervical cancer

**DOI:** 10.1186/s12885-024-11909-3

**Published:** 2024-02-01

**Authors:** Lucely Cetina-Pérez, Julissa Luvián-Morales, Merari Delgadillo-González, Denisse Castro-Eguiluz, Tatiana Galicia-Carmona, Kely Rely, Rita Vaca González, Gabriela Lugo-Martínez, Nadia García-Barrientos, Antonio Nateras

**Affiliations:** 1 Department of Clinical Research, Instituto Nacional de Cancerología, Av. San Fernando No. 22, Sección XVI, 14080 Mexico City, Tlalpan Mexico; 2 MICAELA Program, Instituto Nacional de Cancerología, Mexico City, Mexico; 3 Department of Clinical Research, Investigador por México, Consejo Nacional de Humanidades, Ciencia y Tecnología (CONAHCyT, Instituto Nacional de Cancerología, Mexico City, Mexico; 4 International Healthcare Consultant - CEAHealth Tech, Mexico City, Mexico; 5 Department of Social Work, Instituto Nacional de Cancerología, Mexico City, Mexico; 6 MSD, Mexico City, Mexico

**Keywords:** Cervical cancer, Survival, Demographic characteristics, Prognosis, Epidemiology

## Abstract

**Background:**

In 2020, the highest incidence and mortality from cervical cancer (CC) were detected in low and middle-income countries. CC remains a health problem for women living in them. In Mexico, CC ranks second in cancer incidence and mortality in women. The main characteristics of this population are low income, low educational level, and inadequate medical coverage. The present study characterized the Mexican population by CC, and the sociodemographic variables that impacted overall survival (OS) were identified.

**Methods:**

A retrospective study that included a cohort of patients with a confirmed diagnosis of CC at the Instituto Nacional de Cancerologia between 2003 and 2016. Information was collected on sociodemographic variables related to the disease and OS.

**Results:**

Four thousand six hundred thirty-one patients were included. The median age was 51 years, 78.5% were unemployed, 44.4% lived in a rural/suburban area, 50.8% had a partner when collecting this information, and 74.3% were classified as having low socioeconomic status. Age, living in a rural/suburban area, more advanced stages of the disease, and not receiving cancer treatment were associated with lower OS.

**Conclusion:**

CC continues to affect mainly women with minimal resources, low educational levels, and living in marginalized areas. These characteristics influence the OS. Prevention and timely detection programs, education, and training focused on this population and with broader coverage are required to identify patients with CC at earlier stages.

## Introduction

Cervical cancer (CC) is a health problem occurring mainly in low and middle-income countries, ranking second in incidence and third in cancer mortality among women. It should be added that, globally, in 2020, 88.2% of new cases and 91.4% of CC deaths were detected in these countries [1]. These differences could be due to multiple factors, the lack of coverage of vaccination programs, and the limited efficiency of timely detection strategies to prevent this disease [2].

In Mexico, CC is a health problem, mainly in women of childbearing age. In 2020, approximately 9,429 women were diagnosed with CC, representing 9.2% of cancer cases in women older than 20 years, with CC being the second-highest incidence in Mexican women [1]. The Human Papillomavirus (HPV) is the leading risk factor for the development of CC. The high-risk genotypes are HPV—16, 18, 31, 33, 35, 39, 45, 51, 52, 56, 58, 59, 66, and 68. In Mexico, a positivity of 65% of the HPV-16 and HPV-18 genotypes was attributed to CC patients [3]. Other risk factors for low and middle-income countries CC include beginning sexual activity early, having multiple sexual partners, multiple pregnancies, smoking, using oral contraceptives, and other sexually transmitted diseases. Also, sociodemographic and socioeconomic status (SES) is an associated risk factor for developing CC [4–6].

Approximately 77% of cases in Mexico are detected in locally advanced stages, 16% in early stages, and 7% in advanced stages (metastatic), the histology of the epidermoid type being the most frequent in 80% of cases [7, 8]. The highest mortality occurs in the country's south, mainly in states such as Chiapas, Colima, Baja California, Tabasco, and Morelos [9].

The characteristics of women who develop CC are very peculiar. Some of them are advanced age, low income, being Indigenous, immigrant, having difficulty paying for screening studies, having a low educational level, lack of health insurance, and inadequate medical coverage [10, 11]. In 2003, the "Seguro Popular" (Popular Insurance) was approved in Mexico to provide free health services for some diseases for those without social security. In 2005, CC was incorporated into the interventions with a high catastrophic expenditure, covering screening and treatment for CC in this population, aiming to have better screening and make the treatment of the disease more affordable for the vulnerable population [12].

Although there are studies in Mexico and other countries on the sociodemographic characteristics of women with CC, in Mexico, we seek to demonstrate that factors related to poverty impact survival in a population that is already marginalized, and information on these factors need to be investigated. Therefore, this study aimed to characterize the Mexican population with CC and identify the sociodemographic variables that affect OS.

## Methods

This retrospective study included a cohort of women diagnosed with CC between January 2003 and December 2005 and followed until December 2016 at the Instituto Nacional de Cancerología (National Cancer Institute of Mexico, INCan), Mexico City. Data were collected from the electronic and physical records (2003–2016) at the INCan. Patients ≥ 18 years of age with a confirmed diagnosis of CC through biopsy and a complete record—with information on the clinical variables analyzed in this study— were included. Patients with all histological types were included at any clinical stage and in any functional state.

A total of 4,631 CC patients were included. The stage of the disease was defined according to the criteria of the "International Federation of Gynecology and Obstetrics (FIGO)"—2003 and 2009 [13, 14].

The information from the "Questionnaire for the assignment of socioeconomic status to patients for payment of recovery fees to assign their socioeconomic status" was used to determine the SES. This questionnaire has been used in all the Institutes of the Ministry of Health in Mexico since 1995 [15]. The questionnaire is composed of 5 socioeconomic variables that comprise 100% of the score, distributed as follows: 55% family income, 10% occupation, 10% family expenses, 20% housing, and 5% family health. The points obtained are added once the patients answered this questionnaire in the initial consultation. The SES comprising six levels is assigned, from level 1 (patients with fewer resources) to level 6 (patients with more resources).

This questionnaire included variables that characterized the areas and housing where the patients lived. The variables considered were the state of residence, the place, type of dwelling, housing material, ownership of the home, number of public services included in the residence (water, sewerage, public lighting, paving, garbage collection service, public telephone), intra-domiciliary services (water, electricity, drainage, telephone) and number of people living in a bedroom of the home. In addition, the characteristics of the financial support network were recorded: primary economic provider, academic level of the provider, and the percentage of revenue spent. This percentage was calculated by considering expenses (such as food and services bills) and income (the total amount of money they contribute to all family members).

On the other hand, sociodemographic variables were collected to understand better the SES of the patients, such as age, marital status, educational level, occupation (elementary occupations include simple and routine tasks such as cleaning, washing, and ironing clothes), and religion.

Data associated with the general characteristics of the disease included Karnofsky performance status [16], age, comorbidities, parity, menarche, age of onset of sexual life, approximate time of onset of symptoms and histology, as well as the characteristics of primary cancer treatment in the different stages of the disease.

The study was submitted and approved by the Ethics and Research Committees of the INCan (No. 2021/131).

### Statistical analysis

Descriptive statistics with medians and their minimum and maximum value or frequencies with their respective percentage were used.

The Kaplan–Meier test was used to obtain the cumulative probability of OS at fifteen years, referencing the time elapsed between diagnosis and death or the last visit recorded in the file. The Log-Rank test was used to compare the survival functions in the stratified variables.

For bivariate analysis, the X2 (chi-squared) or Kruskal–Wallis test was used according to the distribution of the variables. The variables that showed a statistical significance or tendency in the bivariate analysis were included in the multivariate analysis. The multivariate analysis was performed using the proportional hazards model (Cox). Only the variables that had a statistical significance or tendency are shown. Interaction terms and proportionality assumptions were evaluated in the final model. Any probability of ≤ 0.05 was considered statistically significant. Two-tailed statistics were used in all cases, and calculations were performed with SPSS version 23 statistical software (IBM Corp., Armonk, NY, USA).

## Results

We analyzed the sociodemographic characteristics of the 4,631 women who met the inclusion criteria (Table 1). According to SES, 38.6% were assigned to Level 1, 35.7% to Level 2, 21.3% to Level 3, 3.2% to Level 4, 0.7% to Level 5 and 0.2% to Level 6. Women with SES levels 1–2 were classified as low SES (74.3%), while those with levels 3–6 were classified as high SES (25.3%). The median age was 51 years, and significantly more patients with high SES were geriatric. Women with low SES were significantly younger at the time of sexual debut and had a median of 4 pregnancies, with a maximum of 22. Most of the women with high SES lived in urban areas, while the more patients with low SES lived in rural areas. No differences were observed regarding marital status; half of the patients had no partner.

**Table 1:** Sociodemographic characteristics of women with cervical cancer according to socioeconomic status

**Variable**	**Total**^b^	**SES 1–2**	**SES 3–6**	p^**^
Study population, n (%)	4631 (100)	3443 (74.3)	1171 (25.3)	
Age, years^a,d^	51 (18–97)	50 (19–97)	52 (18–95)	** < 0.0001**
Geriatric^c^, n (%)	1304 (28.3)	942 (27.4)	362 (30.9)	**0.02**
Age of menarche^a,d^	13 (7–24)	13 (7–24)	13 (7–24)	0.065
Age of sexual debut^a,d^	17 (6–54)	17 (6–54)	18 (6–41)	** < 0.0001**
Parity^a,d^	4 (0–22)	4 (0–22)	5.5 (1–11)	** < 0.0001**
Area of residence^c^, n (%)				
Urban	2565 (44.4)	1646 (47.8)	915 (78.1)	** < 0.0001**
Rural or Suburban	2058 (55.4)	1797 (52.2)	256 (21.9)	
No data	8 (0.2)			
Marital status^c^, n (%)				
Single, widow, divorced, separated	2276 (49.1)	1711 (49.7)	559 (47.7)	0.24
Married, common-law union	2352 (50.8)	1730 (50.3)	612 (52.3)	
No data	3 (0.1)			
Education level^c^, n (%)				
No studies	2240 (48.4)	1778 (51.7)	451 (35.5)	** < 0.0001**
Elementary School or higher	2387 (51.5)	1664 (48.3)	719 (61.5)	
No data	4 (0.1)			
Occupation^c^, n (%)				
No occupation	3634 (78.5)	2735 (79.5)	885 (75.6)	** < 0.0001**
Elementary occupations	839 (18.1)	658 (19.1)	181 (15.5)	
Other	158 (3.3)	49 (1.4)	105 (9)	
No data	3 (0.1)			
Main economic provider^c^, n (%)				
Partner	1831 (39.5)	1359 (39.5)	468 (40)	0.422
Adult progeny	1349 (29.1)	988 (28.7)	358 (30.6)	
Patient	822 (17.7)	625 (18.2)	197 (16.9)	
Other	614 (13.3)	468 (13.6)	146 (12.5)	
No data	15 (0.3)			
% Revenue spent^a,d^	80 (0–1000)	83.3 (0–1000)	57.1 (0–377)	** < 0.0001**

*SES* socioeconomic status

^**^p value represents differences between SES 1–2 vs. SES 3–6. *p* < 0.05 is indicated in **Bold font**

^a^Median (minimum–maximum)

^b^No record of SES was found for 17 patients

^c^Chi-squared test

^d^Mann–Whitney U test

Regarding the level of education, 48.4% of the patients did not receive any education, but 61% of patients with high SES received at least elementary education. Notably, 78.5% of patients had no occupation, which was consistent in both SES groups. The primary economic provider was frequently the partner, followed by adult progeny. Notably, the revenue spent was a median of 80% for all patients and significantly higher for the low SES patients, with a maximum of 1000% (Table 1).

Next, we described the patients’ functional status and characteristics related to the disease and cancer treatment (Table 2). The Karnofsky median score was 90 (minimum 30, maximum 100). The most common comorbidities were high blood pressure (17.3%), which was more frequent in high SES patients, and diabetes (13.1%). Among other comorbidities, renal impairment was reported in 9% of patients. Notably, the onset of symptoms was more than six months in 40% of patients with low SES, compared to 35% of patients with high SES. 36% of patients with high SES had onset of symptoms of less than three months. Most patients (73.4%) were diagnosed at locally advanced stages with squamous-cell carcinoma histology (81.7%). 12.2% of patients with low SES were diagnosed at advanced stages or relapsed or persistent disease, compared to 10.5% of patients with high SES.

**Table 2:** Functional status and characteristics of the disease according to socioeconomic status

**Variable**	**Total**^b^	**SES 1–2**	**SES 3–6**	p^**^
Karnofsky^d,a^	90 (30–100)	90 (30–100)	90 (40–100)	0.056
Comorbidities^c^, n (%)				
High blood pressure	802 (17.3)	554 (16.1)	248 (21.2)	** < 0.0001**
Type 2 Diabetes Mellitus	607 (13.1)	455 (13.2)	152 (13)	0.837
Other	438 (9.4)	279 (8.1)	148 (12.6)	** < 0.0001**
Onset of symptoms^c^, n (%)				
> 6 months	1750 (38.7)	1357 (40.9)	386 (35.1)	**0.003**
3–6 months	1186 (26.2)	868 (26.2)	314 (28.5)	
< 3 months	1497 (33.1)	1093 (32.9)	400 (36.4)	
No data	93 (2.1)			
Characteristics of the disease				
Histology^c^, n (%)				
Squamous-cell carcinoma	3782 (81.7)	2844 (82.9)	924 (79.4)	**0.02**
Adenocarcinoma	537 (11.6)	376 (11)	160 (13.7)	
Other	292 (6.3)	210 (6.1)	80 (6.9)	
No data	20 (0.4)			
Clinical stage^c^, n (%)				
Early (IA1-IB1)	685 (14.8)	485 (14.1)	200 (17.1)	**0.023**
Locally advanced (IB2-IVA)	3399 (73.4)	2539 (73.7)	848 (72.4)	
Advanced (IVB), relapsed or persistent	547 (11.8)	419 (12.2)	123 (10.5)	
Characteristics of cancer treatment				
Early stages^c^, n (%)				
Surgery	368 (53.7)	259 (53.4)	109 (54.5)	0.442
Surgery + adyuvance	163 (23.8)	112 (23.1)	51 (25.5)	
Other	94 (13.7)	73 (15.1)	21 (10.5)	
None	60 (8.8)	41 (8.5)	19 (9.5)	
Locally advanced stages^c^, n (%)				
CRT + BT	1959 (57.6)	1469 (57.9)	489 (57.7)	0.404
Palliative care	11 (0.3)	10 (0.4)	1 (0.1)	
Other	870 (25.6)	656 (25.8)	209 (24.6)	
None	559 (16.4)	404 (15.9)	149 (17.6)	
Advanced, relapsed or persistent^c^, n (%)				
CT	129 (23.5)	100 (23.9)	28 (22.8)	0.818
Palliative care	29 (5.3)	21 (5)	8 (6.5)	
Other	266 (48.6)	207 (49.4)	57 (46.3)	
None	123 (22.5)	91 (21.7)	30 (24.4)	

*SES* socioeconomic status

^*^^*^p value represents differences between SES 1–2 vs. SES 3–6. *p* < 0.05 is indicated in **Bold font**. *CRT* + BT Concomitant chemoradiation therapy followed by brachytherapy, *CT* chemotherapy

^a^Median (minimum–maximum)

^b^No record of SES was found for 17 patients

^c^Chi-squared test

^d^Mann–Whitney U test

Regarding treatment, 53.7% of early-stage CC patients underwent surgery, 57.6% of locally advanced CC patients received CRT followed by BT, and 48.6% of advanced, recurrent, or persistent CC patients received other treatments. No differences in treatment were observed among those with low or high SES (Table 2).

We performed a bivariate and multivariate analysis to identify the prognostic factors associated with OS. Table 3 describes the variables that showed a tendency or were significantly associated with OS. We next performed the multivariate analysis. Having a Karnofsky lower than 90, age younger than 60, living in a rural or suburban area, having SES 1–2, the most advanced stages of the disease, and not receiving cancer treatment were associated with lower OS. Of the factors analyzed, we found that the Karnofsky performance status, clinical stage of the disease, and receiving treatment had the most impact on OS.

**Table 3:** Analysis of prognostic factors associated with OS

Prognostic factor	Bivariate analysis		Multivariate analysis		
	**HR (CI 95%)**	p^*^	**β (SE)**	**HR (CI 95%)**	p*
Karnofsky					
≥ 90	1		-	1	
< 90	2.452 (2.085 – 2.883)	** < 0.0001**	0.616 (0.085)	1.851 (1.566 – 2.188)	** < 0.0001**
Age (years)					
≥ 60	1		-	1	
< 60	0.907 (0.781 – 1.053)	0.201	-0.225 (0.082)	0.798 (0.679 – 0.938)	**0.006**
Marital status					
Single, widowed or divorced	1		-	1	
Married or common-law union	1.133 (0.998 – 1.286)	**0.053**	-0.043 (0.066)	0.958 (0.841 – 1.092)	0.522
Education level					
No studies	1		-	1	
Elementary school or higher	0.865 (0.761 – 0.983)	**0.027**	-0.085 (0.071)	0.918 (0.799 – 1.056)	0.231
Area of residence					
Urban	1		-	1	
Rural o suburban	0.848 (0.745 – 0.965)	**0.013**	-0.316 (0.071)	0.729 (0.635 – 0.838)	** < 0.0001**
SES					
3–6	1		-	1	
1–2	1.049 (0.752 – 1.463)	0.78	0.272 (0.08)	1.315 (1.123 – 1.539)	**0.001**
Clinical stage of CC					
ES	1		-	1	
LAS	5.316 (3.819 – 7.401)	** < 0.0001**	1.635 (0.169)	5.131 (3.683 – 7.149)	** < 0.0001**
AS, recurrent and persistent	19.666 (13.87 – 27.884)	** < 0.0001**	2.707 (0.181)	14.982 (10.512 – 21.353)	** < 0.0001**
Cancer treatment					
Treatment	1		-	1	
No treatment	9.975 (8.141 – 12.222)	** < 0.0001**	1.9 (0.109)	6.686 (5.405 – 8.272)	** < 0.0001**

*CI* confidence interval, *SE* standard error, *SES* socioeconomic status, *CC* cervical cancer, *ES* early stages, *LAS* locally advanced stages, *AS* advanced stages

^*^Risk model by Cox regression. *HR* Hazard Ratio

**Fig. 1:**
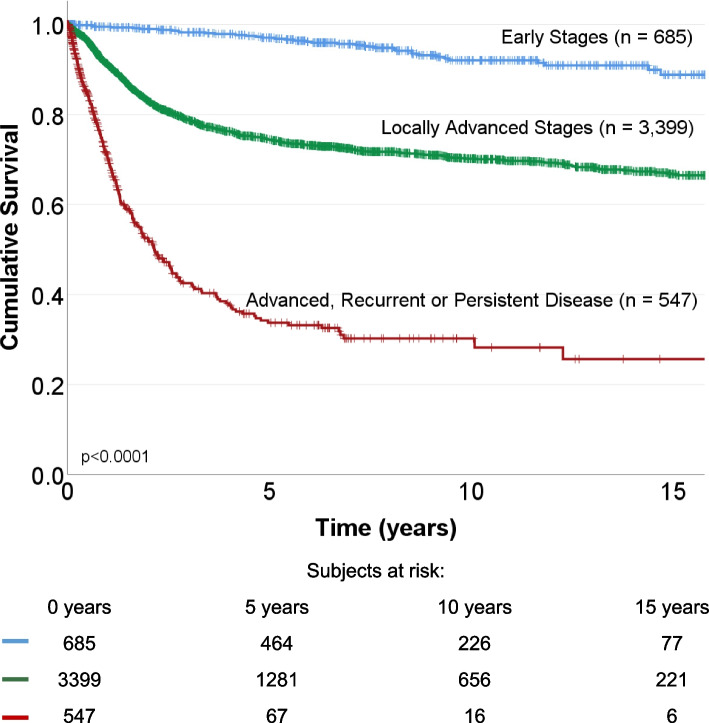
Overall survival of CC patients according to disease status. Top. Kaplan Meier graph shows cumulative survival in early disease (blue), locally advanced disease (green line), and advanced, recurrent, and persistent disease (red line) CC patients, with a follow-up of 15 years. Bottom. According to disease status, the number of CC patients at risk at 5, 10, and 15 years of follow-up

We analyzed the survival of CC patients (Fig. 1). The mean follow-up for OS in this cohort of patients was 4.9 years (0 to 18.9 years), and the mean OS was 13.7 years (95% CI: 13.4–14). 20.7% of patients died from CC, 2.3% died from other causes, 20.6% were alive, and 56.4% were lost to follow-up. A significant difference in OS was observed between stages.

We analyzed the patients lost to follow-up. 20.2% of the lost patients did not even begin treatment; these were over 50 years old, with a Karnofsky functional status score of less than 80, were diagnosed with more advanced disease stages, were economically dependent, had no partner, and their income expenditure exceeded 80%.

## Discussion

Diagnosis of early-stage cervical cancer is imperative to increase the chances of curative treatment and survival. Sociodemographic characteristics may play a role in the timing of diagnosis and subsequent care of women with CC. Explanations for socioeconomic differences in survival are not well documented. However, the possible underlying causes can be separated into factors related to the tumor, the patient, and the healthcare system [17].

Few studies relate the SES of CC patients with OS. This study aimed to characterize the CC population and identify the sociodemographic factors associated with OS. Our study observed that patients with CC belong to the less-benefited population and that factors such as functional status, age, living in an urban area, having a lower SES, having an advanced stage of the disease, and not receiving cancer treatment increased the risk of mortality.

Some past attempts to explain social differences in cancer survival have focused on differences in disease stage at diagnosis [18]. It has been estimated that between 68 and 77% of CC cases in Mexico are diagnosed with LAS [7, 8]. Other reports have similar findings to our study, where OS in the advanced, relapsed, and persistent stages is lower than in the early stages [8, 19, 20]. We confirm that the clinical stage is the strongest predictor of all clinical prognostic factors, and a lower SES relates to a more advanced disease [21]. Studies show that women with lower SES are more likely to be diagnosed at an advanced stage [22, 23]. Because there is a lack of health insurance and factors related to the health care system, the detection of CC is likely to be diagnosed in more advanced stages [24–26]. This may be also related to less frequent visits to the doctor and lack of CC screening [27]. On the other hand, socioeconomic differences may impact diagnostic quality. Patients with lower SES may be misdiagnosed with localized disease while actually having a more advanced disease [24].

We observed that the functional status of patients plays an essential role in predicting their prognosis. A Karnofsky score below 90 was associated with a lower OS, which was also reported in a previous study [28]. A decrease in performance status may be linked to increasing age, as studies have shown that individuals above 65 are more likely to have a Karnofsky score below 90 [28]. Our study found that patients over 60 had a lower OS. Several other studies have reported that older age is associated with lower OS, regardless of the disease stage. For instance, women aged 50 to 69 have a risk index of 1.46, while those over 70 have a risk index of 2.87, making them more likely to receive palliative treatment or no treatment at all. This finding is consistent with the results of our study [28, 29].

Previously, it was demonstrated that comorbidities were associated with higher mortality after surgical treatment [30, 31]. More so, the presence of comorbidities is a factor that influences the decision of cancer treatment, which may be related to OS. A retrospective study observed that comorbidities were a significant predictor of external RT reinforcement, associated with lower OS and more significant toxicity in LAS patients [32]. In the present study, comorbidities had no association with OS; a possible explanation is a sub-registry of this information, mainly in physical records.

Education could be a factor related to opportune diagnosis and treatment for CC. It was found that women with no education needed help understanding CC, and their ability to cope with particular situations was impaired; consequently, the patients did not adequately comply with medical follow-up [33]. A study in Ethiopia described that having a lower educational level, being young, and being informed through mass media were associated with insufficient knowledge of the disease [34]. In other Latin-American countries limited education was a factor related to CC mortality [27]. Although low education was frequent in our population, it did not impact OS.

Evidence suggests that social support leads to better OS [35, 36], possibly because better social support leads to timely seeking of an appropriate diagnosis and treatment [30]. In this study, just over half of the patients who attended had a partner whose key role was economic provider. However, having a partner was not a prognostic factor for OS. Social support is less prevalent among disadvantaged or low-income groups, and if such factors impact survival, they could contribute to socioeconomic differences [37].

According to the results, 16.8% of women lived in rural areas. In other populations, women who live in rural areas tend to delay their medical care more frequently than those who live in urban areas [31, 38–40]. A study in Colombia reported higher mortality in women living in rural areas [41]. In the southern region of Mexico, in the most marginalized states, mortality rates from CC are higher than in the center or north of the country. It should be added that, for years, the decrease in the mortality rate has been more significant in the center than in other regions [8, 19]. The differences in mortality between areas may indicate the ineffectiveness of the current health programs for reducing CC mortality throughout the country [19]. Surprisingly, we found that patients in rural or suburban areas had higher OS than those in urban areas. Explanations for this finding may be related to the urban lifestyle, which includes the increased levels of psychological stress, sedentarism, obesity, smoking, and alcohol consumption that could impact OS [42].

An important finding was that receiving cancer treatment was associated with higher OS, which justifies the need to have all the infrastructure, human, and material resources to offer a quality therapeutic option to CC patients.

Among the limitations of this study is that information was collected retrospectively, and updates in the format to gather the information made it impossible to assign the SES to some patients. Another limitation was the high number of follow-up losses (57%). This patient loss is in line with reports from another study, which mentioned that about 47% of patients with CC in Mexico only attend one consultation and subsequently abandon their follow-up [8]. Finally, there was no information from 2 states (Coahuila and Guadalajara) of the 32 that comprise the country, and the number of cases per state ranged from 1 to 1,496.

Among the strengths of this study is the analysis of various sociodemographic variables that impact OS in CC patients. Likewise, there were few excluded cases (3%), which makes the results applicable to the clinical setting.

## Conclusions

Belonging to a low SES, having a low educational level, and living in marginalized areas remain a constant in Mexican women with CC. Among the sociodemographic factors associated with OS are functional status, age, area of residence, and SES. Since the clinical stage of the disease and treatment are the most decisive factors related to OS, prevention and detection programs with broader coverage are required to identify patients with CC in earlier stages to offer treatments that increase OS.

## Data Availability

The datasets generated and analyzed during the current study are available from the corresponding author upon reasonable request.

## Abbreviations

AS: Advanced clinical stages
CC: Cervical Cancer
ES: Early clinical stages
LAS: Locally advanced stages
SES: Socioeconomic Status
CRT + BT: Concomitant chemoradiation therapy followed by brachytherapy
CT: Chemotherapy
RT: Radiotherapy
OS: Overall survival
